# Efficacy of pentosan polysulfate in *in vitro* models of lysosomal storage disorders: Fabry and Gaucher Disease

**DOI:** 10.1371/journal.pone.0217780

**Published:** 2019-05-31

**Authors:** Andrea N. Crivaro, Juan M. Mucci, Constanza M. Bondar, Maximiliano E. Ormazabal, Romina Ceci, Calogera Simonaro, Paula A. Rozenfeld

**Affiliations:** 1 IIFP, Universidad Nacional de La Plata, CONICET, Facultad de Ciencias Exactas, Departamento de Ciencias Biologicas, La Plata, Argentina; 2 Icahn School of Medicine at Mount Sinai, New York, New York, United States of America; Weizmann Institute of Science, ISRAEL

## Abstract

Gaucher and Fabry diseases are the most prevalent sphingolipidoses. Chronic inflammation is activated in those disorders, which could play a role in pathogenesis. Significant degrees of amelioration occur in patients upon introduction of specific therapies; however, restoration to complete health status is not always achieved. The idea of an adjunctive therapy that targets inflammation may be a suitable option for patients. PPS is a mixture of semisynthetic sulfated polyanions that have been shown to have anti-inflammatory effects in mucopolysaccharidosis type I and II patients and animal models of type I, IIIA and VI. We hypothesized PPS could be a useful adjunctive therapy to inflammation for Gaucher and Fabry diseases. The objective of this work is to analyze the *in vitro* effect of PPS on inflammatory cytokines in cellular models of Gaucher and Fabry diseases, and to study its effect in Gaucher disease associated *in vitro* bone alterations. Cultures of peripheral blood mononuclear cells from Fabry and Gaucher patients were exposed to PPS. The secretion of proinflammatory cytokines was significantly reduced. Peripheral blood cells exposed to PPS from Gaucher patients revealed a reduced tendency to differentiate to osteoclasts. Osteoblasts and osteocytes cell lines were incubated with an inhibitor of glucocerebrosidase, and conditioned media was harvested in order to analyze if those cells secrete factors that induce osteoclastogenesis. Conditioned media from this cell cultures exposed to PPS produced lower numbers of osteoclasts. We could demonstrate PPS is an effective molecule to reduce the production of proinflammatory cytokines in *in vitro* models of Fabry and Gaucher diseases. Moreover, it was effective at ameliorating bone alterations of *in vitro* models of Gaucher disease. These results serve as preclinical supportive data to start clinical trials in human patients to analyze the effect of PPS as a potential adjunctive therapy for Fabry and Gaucher diseases.

## Introduction

Lysosomal diseases are a group of more than 50 genetic disorders caused by pathogenic mutations in genes associated to lysosomal proteins. Various lysosomal disorders are due to enzyme deficiencies leading to specific substrate accumulation within lysosomes. Gaucher and Fabry diseases are the most prevalent sphingolipidoses. Although the primary cell defect is completely known the pathophysiology is not completely uncovered. When disease status initiates at the cellular level, with substrate accumulation as the primary defect, secondary responses are triggered. These secondary effects are new, unique and may be independent of the primary defect [1].

The concept of the existence of chronic stimulation of the immune system in lysosomal disorders has been introduced more than 3 decades ago. Inflammation is a hallmark in many lysosomal disorders, characterized by high levels of proinflammatory cytokines such as TNFα, IL1β, IL6 [2]. These cytokines are secreted by innate immune cells when their toll-like receptors or NOD-like receptors recognize molecular patterns associated to pathogens or danger signals (DAMPs) [3]. There is evidence that accumulated lysosomal substrates could behave as DAMPs [4,5,6]. Alternatively, cell or tissue stress produced in response to deposits could be the source of endogenous molecules recognized as DAMPs.

Inflammation is a quick and acute natural response of the immune system activated upon by the presence of a pathogen or stress signals. It is self-limiting once the trigger is no longer present. On the other side, chronic inflammation is a disease state, and it is established if danger signals cannot be eliminated. Chronic inflammation is generally a silent and slow process [7], and tissue pathogenesis is not evident until there is irreversible damage with clinical sequelae. This situation could be the case in lysosomal disorders, where there is a continuous accumulation of substrates [8].

Fabry disease is caused by pathogenic mutations in *GLA* gene causing enzymatic deficiency of alpha-galactosidase A leading to accumulation of globotriaosylceramide (Gb3) and lyso-Gb3 not limited to lysosomes but also in plasma membranes and caveolae of endothelial cells. Clinical manifestations may appear in childhood, and affection of target organs, kidney, heart and brain, occurs in 3^rd^-4^th^ decades of life. It has been suggested that Gb3 accumulation dysregulates endothelial NO synthase leading to increased oxidative stress and reactive oxygen species (ROS) resulting in Fabry’s cardiovascular-renal disease. Production of high levels of proinflammatory cytokines are observed in Fabry patients. This effect could be produced as a result of exposure to high levels of Gb3 to TLR4 in immune cells [4].

Gaucher disease is an autosomal recessive disorder due to deficient activity of the lysosomal enzyme glucocerebrosidase (GCase) produced by pathogenic mutations in *GBA1* gene. This deficiency leads to accumulation of glucosylceramide mainly in macrophages. The most common phenotype is type I GD (GD1) consisting of visceral, hematological and skeletal alterations [9]. It was recently shown that the substrate glucosylceramide is an endogenous ligand for the receptor Mincle present in innate immune cells. This interaction induced the production of inflammatory cytokines such as TNFα and IL-1β [6]. Abnormal bone manifestations are the most debilitating symptoms for Gaucher patients, despite available treatment. It is well known that the skeletal system is affected by immune system, and inflammation may have a negative impact in bone homeostasis targeting osteoclasts, osteoblasts and osteocytes. [10].

For these two sphingolipidoses, specific therapies such as enzyme replacement therapy (ERT) have been introduced. Significant degrees of amelioration occur in patients; however, restoration to complete health status is not always achieved [11]. This depends on different aspects related to onset of disease in the patient, severity, onset of therapy, and secondary responses. With respect to chronic inflammation little is known if there is a beneficial effect of ERT on this immune response because many studies have shown no changes in cytokines of treated patients [4, 12]. For this reason, the idea of an adjunctive therapy that targets inflammation may be a suitable option for patients.

In the last years, several works have been published in relation to cartilage and bone pathology in mucopolysaccharidoses (MPS) where inflammation was shown to be a key factor in pathogenesis [13]. A compound called pentosan polysulphate (PPS) was proposed as therapy in MPS models [14] and showed marked improvements in clinical behavior in addition to a reduction of glycosaminoglycans [15, 16]. Based on these beneficial effects, clinical trials were undertaken and are ongoing in MPS patients [17].

PPS is a mixture of semisynthetic sulfated polyanions that is approved by the FDA as an oral medication for the treatment of interstitial cystitis, an inflammatory-like disease [18]. The mechanism of anti-inflammatory action of PPS is not clear, but there is a report showing it decreases NFkB activation [19].

Given the positive effect of PPS to reduce inflammation in mucopolysaccharidosis type I and II patients and animal models of type I, IIIA and VI, we hypothesized PPS could be a useful adjunctive therapy to inflammation in other LSD, such as Gaucher and Fabry diseases. The objective of this work is to analyze the *in vitro* effect of PPS on inflammatory cytokines in cellular models of Gaucher and Fabry diseases, and to study its effect in Gaucher disease associated *in vitro* bone alterations.

## Materials and methods

### Ethics statement

This study was approved by the Ethical Committee of IBYME (Instituto de Biología y Medicina Experimental, CONICET, Argentina) according to provisions of the Declaration of Helsinki in 1995. Human PBMCs were isolated from patients and healthy blood donors in accordance with the guidelines provided by the ethical committee. The nature and purpose of the study were explained to all volunteers and all patients gave their written informed consent prior to participation in this study. In the case of minors/children written informed consent was obtained from the next of kin, caretakers, or guardians.

### PBMC isolation and culture

Peripheral blood samples from 20 Fabry (F) and 14 Gaucher (G) patients or healthy controls were collected. In the case of Gaucher patients samples were obtained 24 h before ERT infusion. For Fabry studies, 15 samples were obtained from treatment naïve patients. Blood was taken by venipuncture in heparin as anticoagulant and immediately processed. Mononuclear cells from whole blood (PBMC) were isolated by Ficoll Hypaque (Sigma, St Louis,MO, USA) gradient separation.

For cytokine levels, chitotriosidase activity and indirect osteoclast differentiation measurements Fabry and Gaucher cells (2x10^6^ cells/ml) were cultured in the presence of 5 μg/ml of PPS for 72 h and conditioned media (CM) was harvested.

For direct osteoclastogenesis assays, PBMC from Gaucher patients or healthy donors were seeded at 5 × 10^5^ cells/ml and cultured at 37°C in 5% CO2 atmosphere in α-minimum essential medium (α-MEM) supplemented with 2 mM L-glutamine, 10% heat inactivated fetal bovine serum (Gibco-BRL, Life technologies, Grand Island, NY), 100 U of penicillin per ml and 100 μg of streptomycin per ml (complete media) and 30 ng/ml of recombinant human macrophage colony stimulating factor (M-CSF) (R&D, Minneapolis, MN, USA) for 14 days replacing the media every 72 h. To identify osteoclasts, cells were fixed in 4% paraformaldehyde and stained for tartrate resistant acid phosphatase (TRAP; Sigma Aldrich, St Louis, MO, USA). TRAP positive multinucleated (more than 3 nuclei) cells were defined as osteoclasts, and the number was determined by microscopic counts on a whole well.

### Generation of Fabry disease *in vitro* model

Macrophage differentiation was carried out as previously described [4]. Briefly, PBMC obtained from healthy donors buffy coats were washed with sterile pyrogen-free saline, centrifuged at 100 g for 10 min, and resuspended in AIM-V medium. Fifty ×10^6^ cells were seeded in 75 cm2 culture flasks and cultured for 1 h at 37°C and 5% CO2. Non-adherent cells were removed by washing twice with warm saline, and remaining cells were cultured in AIM-V for 6 days with the addition of 30 ng/ml recombinant human M-CSF (R&D Systems, Minneapolis, MN, USA), to stimulate their differentiation to macrophages (MΦ). Obtained cells were gently resuspended in AIM-V, seeded in 12-well culture plates (5×105 cells/ well) and cultured for 24 h in the presence of 20 μM Gb3 (Matreya, Pleasant Gap, PA, USA) and/or 200 μM 1-deoxygalactonojirimycin (DGJ, Sigma). For dose response experiments, 24 h culture media was replenished with fresh media containing Gb3, DGJ and 2, 5 or 10 μg/ml of PPS and 72 h followed by harvesting of the supernatant. In the case of kinetics experiments, after the initial 24 h media was replenished with fresh media containing Gb3, DGJ and 5 μg/ml of PPS and the supernatant was harvested 24, 48 or 72 h later.

### Cell line cultures

The murine osteocyte cell line MLO-Y4 was cultured in α-minimum essential medium (α-MEM media, Gibco-BRL, Life Technologies, Grand Island, NY) supplemented with 2.5% of FBS, 2.5% of bovine calf serum (Gibco-BRL), 100 U of penicillin per ml and 100 μg/ml of streptomycin, at 37°C in a 5%CO2 atm for 72 h in the presence or absence of conduritol- β-epoxide (CBE) 500 mM (Matreya) and 5 μg/ml of PPS and the conditioned media was harvested.

Murine osteoblasts cell line MC3T3 was cultured at 37°C in 5% CO_2_ atm in α-MEM supplemented with 2 mM L-glutamine, 10% heat inactivated fetal bovine serum (Gibco-BRL, Life Technologies, Grand Island, NY), 100 U of penicillin per ml and 100 μg of streptomycin per ml, in the presence or absence of 5 μg/ml of PPS. For conditioned media preparation supernatants were harvested 72 h after PPS addition. For osteoblast activity assays media was supplemented with osteogenic supplements, 1 mM sodium glycerophosphate, 50 mM L ascorbate and 10^−8^ M dexamethasone (all Sigma Aldrich), for 14 days with changes of medium and PPS every 3–4 days.

### Indirect osteoclastogenesis studies

For testing induction of osteoclast formation by the addition of CM from PBMC, THP-1 cells were seeded at 5 × 10^5^ cells/ml and cultured at 37°C in 5% CO2 atmosphere in complete media and 30 ng/ml of recombinant human M-CSF for 72 h. Non- adherent cells were washed out and adherent cells were used for the osteoclast formation assays.

For CM derived from mice cells, bone marrow-derived monocytes from C57Bl/6 mice were seeded at 5 × 10^5^ cells/ml and cultured at 37°C in 5% CO_2_ atm complete media supplemented with 30 ng/ml of recombinant murine macrophage colony stimulating factor (M-CSF) (R&D, Minneapolis, MN, USA) for 48 h. Non-adherent cells were washed out and adherent cells were used for the osteoclast formation assays. For all the assays the adherent cells were cultured in CM and complete medium in a 1:1 ratio supplemented with M-CSF for 7 days replacing the media every 48 h.

To identify osteoclasts, cells were fixed in 4% paraformaldehyde and stained for tartrate resistant acid phosphatase (TRAP; Sigma Aldrich, St Louis, MO, USA). TRAP positive multinucleated (more than 3 nuclei) cells were defined as osteoclasts, and the number was determined by microscopic counts on a whole well.

### CHIT activity determination

CHIT activity was assessed in DBS (dried blood spot) and supernatant as follows: 3mm diameter circle from DBS filter paper or 20 μl supernatant was placed into a well of a black microplate and 40 μl of 0.25M sodium acetate buffer pH = 5.5 and 40 μl of 0.19 mM 4-metilumbeliferil β-D-N-N′-N″- triacetylchitotrioside (Sigma) were added. After an incubation for 30 min at 37°C, the stop solution (220 μl of 0.1 mol/l ethylenediamine, pH = 11.4) was added. The fluorescence of the product (excitation 365 nm; emission 450 nm)was measured on a Twinkle LB 970 fluorometer (Berthold Technologies, BadWild-bad, Germany). A standard curve of 4-methylumbelliferone (Sigma, Saint Louis, MO, USA) was used to extrapolate fluorescence counts to moles of enzymatic product. Enzymatic activity was expressed as micromoles of 4-methylumbelliferone produced per liter per hour.

### Cytokine measurement

Cytokine levels in the supernatant fraction were quantified by capture ELISA (BD Biosciences) following the manufacturer instructions.

### Glycolipid measurement

Plasma globotriaosylsphingosine (lyso-GB3) was measured by liquid chromatography tandem mass spectrometry (LC-MS/MS) using a published method with minor modifications [20]. Dimethyl psychosine was used as internal standard. Acquity UPLC BEH C18 column (2.1 mm X 50 mm with 1.7 μm particle size) was used for chromatography. Plasma glucosylsphingosine (lyso-GL1) was measured by a published LC-MS/MS method with minor modifications [21]. D5-glucosylsphingosine was used as internal standard. Lyso-GL1 is separated from its isomer galactosylsphingosine (psychosine) using Acquity UPLC BEH amide column (2.1 mm X 100 mm with 1.7 μm particle size). Agilent 6490 triple quadruple mass spectrometer coupled with Agilent 1290 liquid chromatography system was used for both analyses. Measurements were performed in cell pellets and culture supernatants as indicated in each figure.

### Quantification of mineralization by Alizarin Red S (ARS)

Monolayers in 6-well plates (10 cm2/well) were washed with PBS and fixed in 10% (v/v) formaldehyde (Sigma–Aldrich) at room temperature for 15 min. The monolayers were then washed twice with excess dH_2_O prior to addition of 1 mL of 40 mM ARS (pH 4.1) per well. The plates were incubated at room temperature for 20 min with gentle shaking. After aspiration of the unincorporated dye, the wells were washed four times with 4 mL dH_2_O while shaking for 5 min. 800 μL 10% (v/v) of acetic acid was added to each well, and the plate was incubated at room temperature for 30 min with shaking. The monolayer, now loosely attached to the plate, was then scraped from the plate with a cell scraper (Fisher Life Sciences) and transferred with 10% (v/v) acetic acid to a 1.5-mL microcentrifuge tube. After vortexing for 30 s, the slurry was overlaid with 500 μL mineral oil (Sigma-Aldrich), heated to exactly 85°C for 10 min, and transferred to ice for 5 min. The slurry was then centrifuged at 20,000 g for 15 min and 500 μL of the supernatant was removed to a new1.5-mL microcentrifuge tube. Then 200 μL of 10% (v/v) ammonium hydroxide was added to neutralize the acid. In some cases, the pH was measured at this point to ensure that it was between 4.1 and 4.5. Aliquots (150 μL) of the supernatant were read in triplicate at 405 nm.

### Sirius Red

Osteoblasts cultured on 24 well culture plates were washed 3 times with PBS and fixed using 250 μl of Bouin fluid for 1 h. Bouin fluid was freshly prepared prior to use by mixing 15 ml of saturated picric acid with 5 ml of formaldehyde 35% v/v. After fixation, cells were washed 3 times with excess of distilled water and allowed to dry for several minutes. 1 ml of a 0.1% Sirius Red (Sigma-Aldrich) in picric acid solution was added and incubated at room temperature for 18 h with gentle shaking. After the incubation, cells were washed 5 times with 0.01 N HCl to remove excess dye. For quantification 200 μl of 0.1 N NaOH were added and incubated for 30 min with shaking to dissolve the dye and the solution absorbance was measured at 550 nm.

### Statistical analysis

Statistical analyses were performed using GraphPad Prism 5.0 program applying t-test. Data are presented as mean±SD. Experiments involving cells lines were carried out with an n = 5 and performed 3 independent times.

## Results

### Effect of PPS on cytokine production by Fabry macrophages *in vitro* chemical model

**Fig 1:**
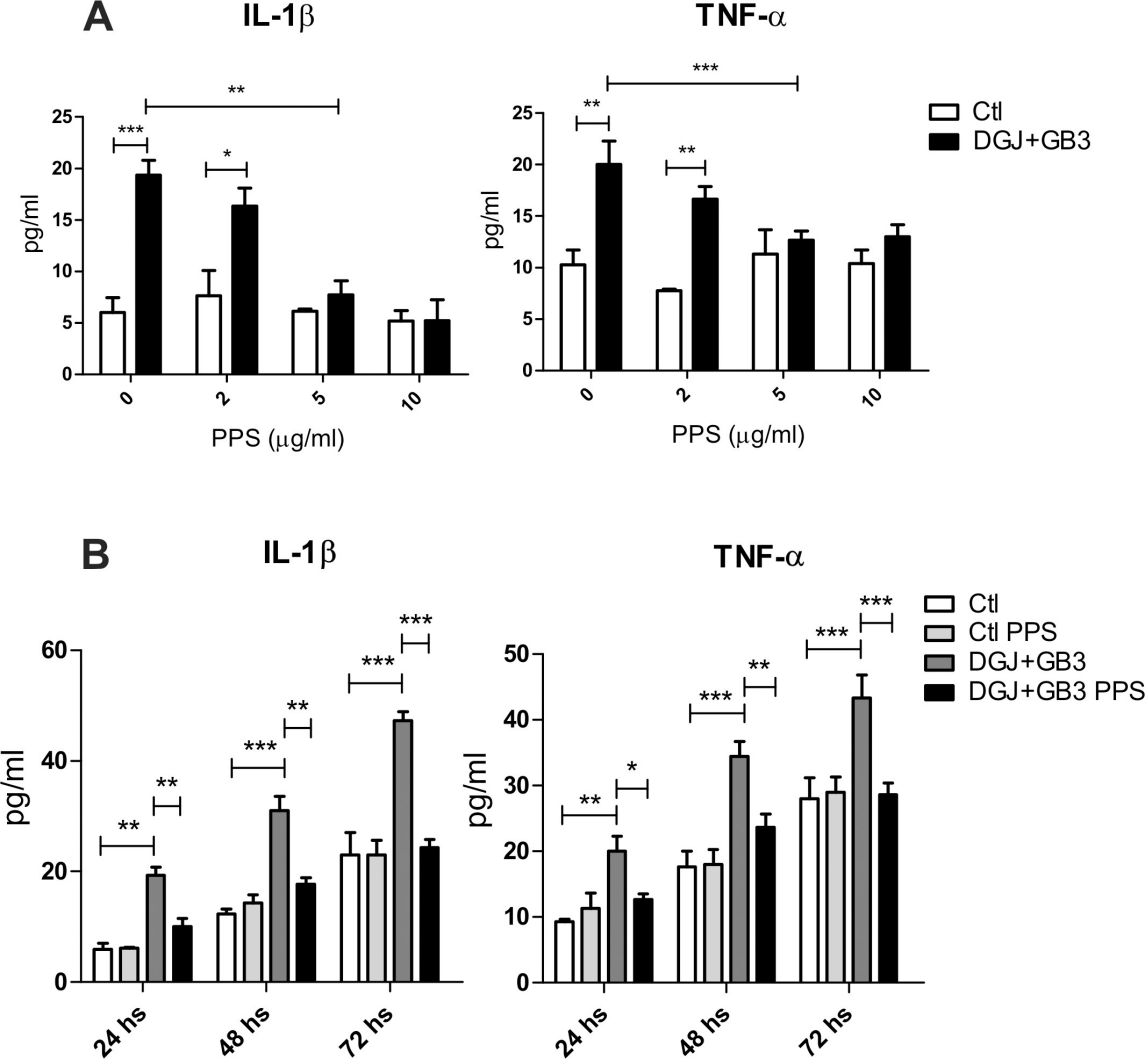
Fabry macrophages model with Gb3/DGJ: dose response (A) and kinetics (B). Based on this study, 5 μg/ml is the minimum dose responsible for cytokine reduction. Data is shown as the mean±SD with n = 5 one representative experiment of 3 performed. *p<0,05 **p<0,01 ***p<0,001 t-test.

Previously, we were able to reproduce the production of proinflammatory cytokines in Fabry disease by the use of an *in vitro* chemical model of macrophages treated with Gb3 and DGJ [4]. We decided to use this model to check the proof of concept that PPS is able to inhibit cytokine secretion in Fabry disease. A dose-response assay was carried out with concentrations of 2, 5 and 10 μg/ml of PPS in order to test efficacy and minimum effective dose. Addition of PPS to this cell model significantly reduced the production of IL-1β and TNFα to control levels. As can be seen in Fig 1A, the dose of 5 μg/ml is the minimum dose responsible for this effect.

To evaluate the best incubation time for PPS treatment we carried out a kinetics assay. Macrophages were cultured in the presence of Gb3 and DGJ as previously and a dose of 5 μg/ml of PPS for 24, 48 and 72 hours. As seen in Fig 1B, a reduction in TNF-α and IL-1β was observed from 24 h of treatment achieving the highest differences in the 72 h time point. Based on these results we chose the 5 μg/ml dose for 72 h of PPS treatment for the remainder of the experiments performed.

### Cytokine determination in culture supernatants from Fabry patients

**Fig 2:**
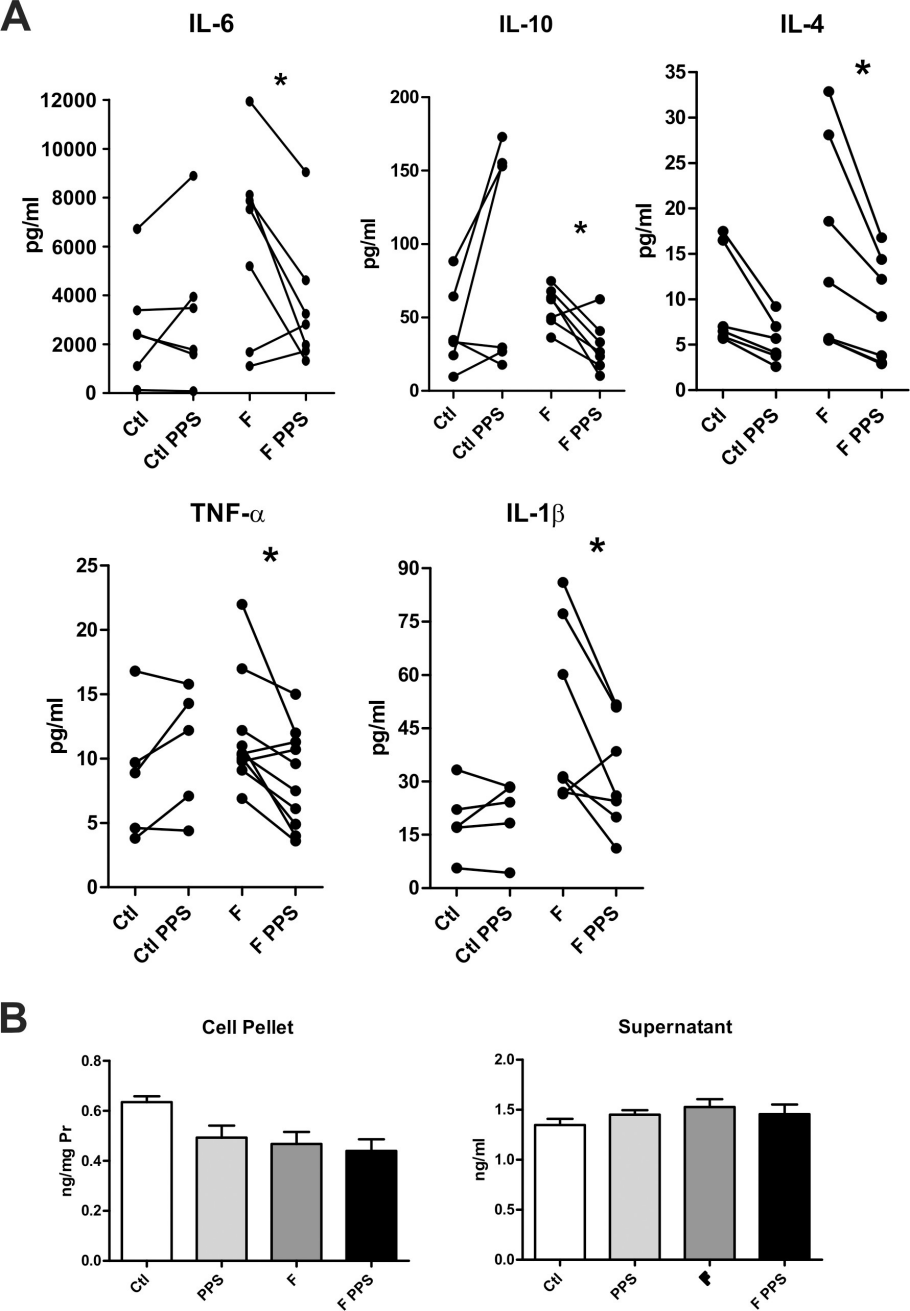
Cytokine determination in culture supernatants from naïve Fabry patients (F) and healthy controls (Ctl), in the presence or not of PPS (2A). Lyso Gb3 levels in cell homogenates and culture supernatants (2B) from naïve Fabry patients and controls with or without PPS. Data is shown as the mean±SD. *p<0,05 t-test.

Having demonstrated a positive effect of PPS in the chemical model, we decided to analyze the response in mononuclear cells from Fabry patient’s. A significant reduction in the secretion of cytokines IL-1β, TNFα, IL-6, IL-4 and IL-10 was revealed when PBMC from Fabry patients were incubated with PPS (Fig 2A).

In order to discriminate if the effect of PPS was due to its well-known anti-inflammatory properties or an unknown effect on glycolipid deposits we measured LysoGb3 levels in cell homogenates and supernatants, with and without PPS. As can be seen in Fig 2B, PPS treatment did not change LysoGb3 levels.

### Cytokine and chitotriosidase determination in culture supernatants from Gaucher patients

**Fig 3:**
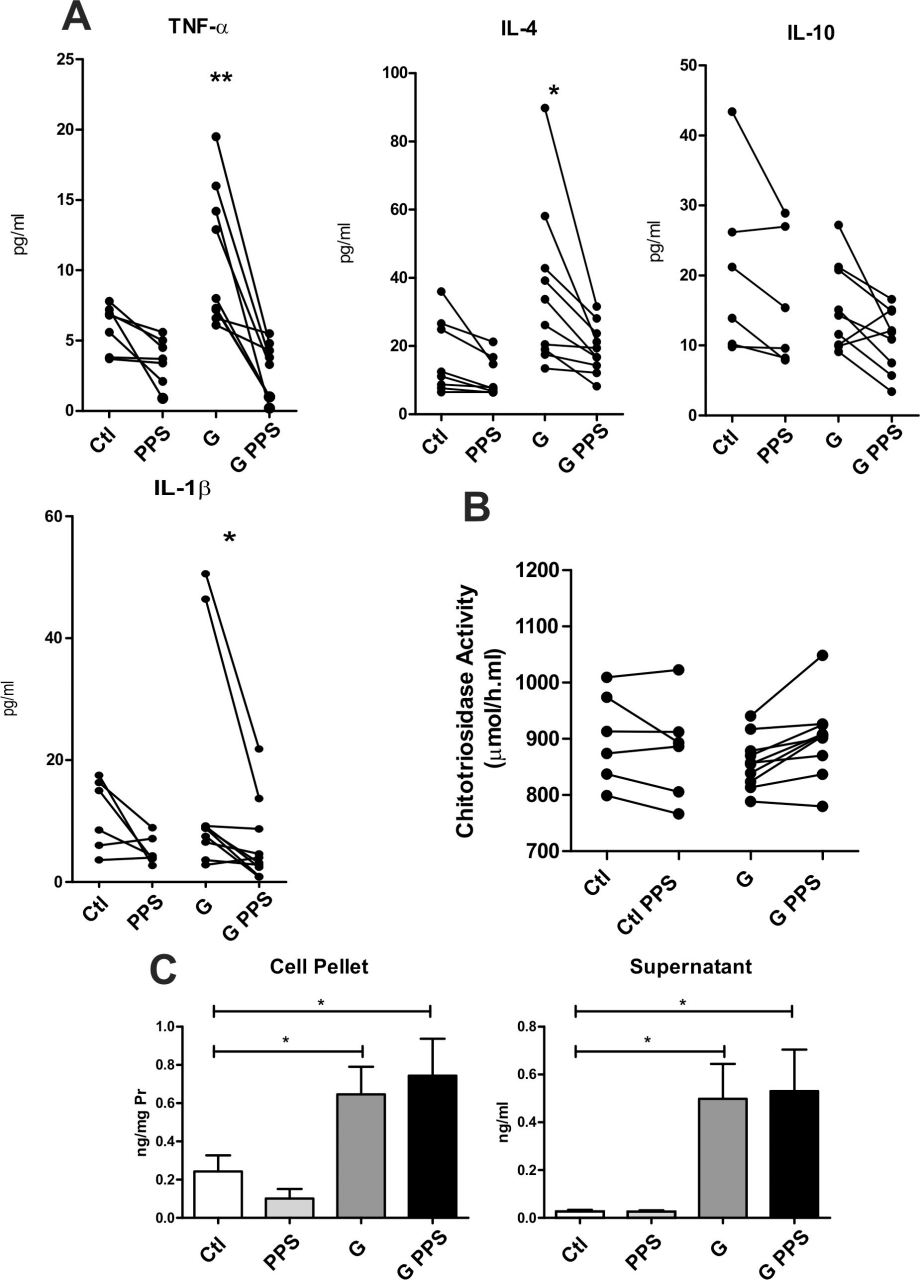
Cytokine (3A) and chitotriosidase (3B) determination in culture supernatants from Gaucher patients (G) or healthy donors (Ctl), in the presence or not of PPS. Lyso GL1 levels in cell homogenates and culture supernatants (3C) from Gaucher patients and controls with or without PPS. Data is shown as the mean±SD. *p<0,05 **p<0,01 t-test.

Given the positive effect observed in Fabry disease cultures, we tested the effectiveness on another sphingolipidosis, Gaucher disease. PBMC from Gaucher patients treated with PPS secrete significantly less quantities of IL-1β, TNFα and IL-4 (Fig 3A). In Gaucher disease, the biomarker chitotriosidase correlates with clinical severity and it is a good marker for follow-up of ERT. For this reason we decided to measure this biomarker. No difference was observed in chitotriosidase measurement from PBMC treated with PPS as compared to non-treated cultures (Fig 3B).

As above, we assayed levels of LysoGL1 in Gaucher patients cells with or without PPS treatment. No differences in LysoGL1 were detected because of PPS treatment (Fig 3C).

### Osteoclastogenesis studies

In the era of ERT, the skeletal manifestations represent the main obstacles for Gaucher patients [22]. It is well recognized that an inflammatory environment alters bone homeostasis [23]. Studies have suggested an imbalance in bone homeostasis in GD where osteoclastogenesis and bone resorption would be increased and bone formation would be reduced [24,25]. Both processes could be triggered by high levels of proinflammatory cytokines in the bone microenvironment. For this reason we questioned if PPS could have a beneficial effects on bone homeostasis.

**Fig 4:**
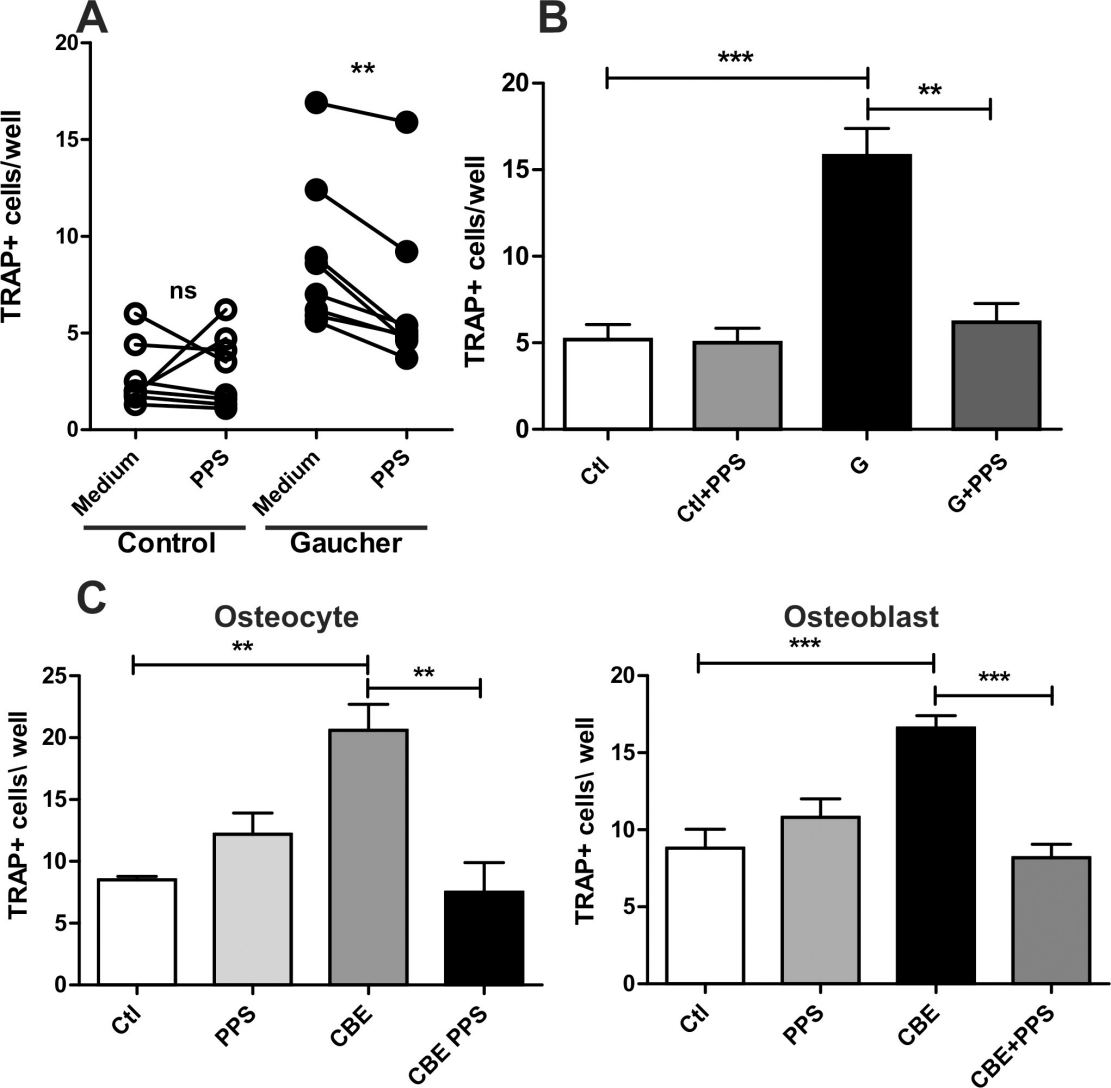
Direct osteoclastogenesis from PBMC (A). Indirect osteoclastogenesis mediated by PBMC supernatant (B), osteoblasts and osteocytes (C). Data is shown as the mean±SD with n = 5 one representative experiment of 3 performed. **p<0,01 ***p<0,001 t-test.

In a previous work we have shown that PBMC from Gaucher patients have higher tendency to differentiate into functional osteoclasts as compared to normal controls when cultured with M-CSF [12]. We performed the same experiments in the presence of PPS. A significant reduction in osteoclast differentiation was observed for GD patient samples while no difference was present in healthy control samples (Fig 4A). We also showed that THP-1 derived osteoclast precursors cultured in the presence of CM from PBMC of GD patients had higher differentiation to active osteoclasts and that this effect was mediated, at least in part, by TNF-α. As showed in Fig 3A PPS reduced TNF-α release from PBMC of GD patients therefore we decided to test if PPS had an effect on osteoclastogenesis. We cultured PBMC from GD patients and healthy controls in the presence of PPS for 72 h and harvested the CM. The CM was used in osteoclast differentiation assays using THP-1 derived osteoclast precursors. Conditioned media from PPS treated GD PBMC induced significantly less osteoclast differentiation that CM from untreated cells, while no difference was observed when control PBMC were used (Fig 4B).

Osteocytes are long-lived cells that derive from osteoblasts. Both cell types constitute a source of RANKL, the main inducer of bone resorption [26]. In a previous work we showed that CM from CBE treated osteocytes induced higher osteoclast differentiation that control CM [25]. In the present work we questioned if PPS could reduce this effect. Therefore, we cultured MLO-Y4 cells (an osteocyte cell line) and MC3T3 cells (an osteoblast cell line) in the presence of CBE and/or PPS for 72h and conditioned media were harvested. The CM was used for osteoclast differentiation using osteoclast precursors derived from bone marrow precursors. As shown in Fig 4C CM from CBE treated osteocytes and osteoblasts generated higher osteoclastogenesis that control CM while PPS treatment reduced this effect to control levels.

### Bone formation by osteoblasts

**Fig 5:**
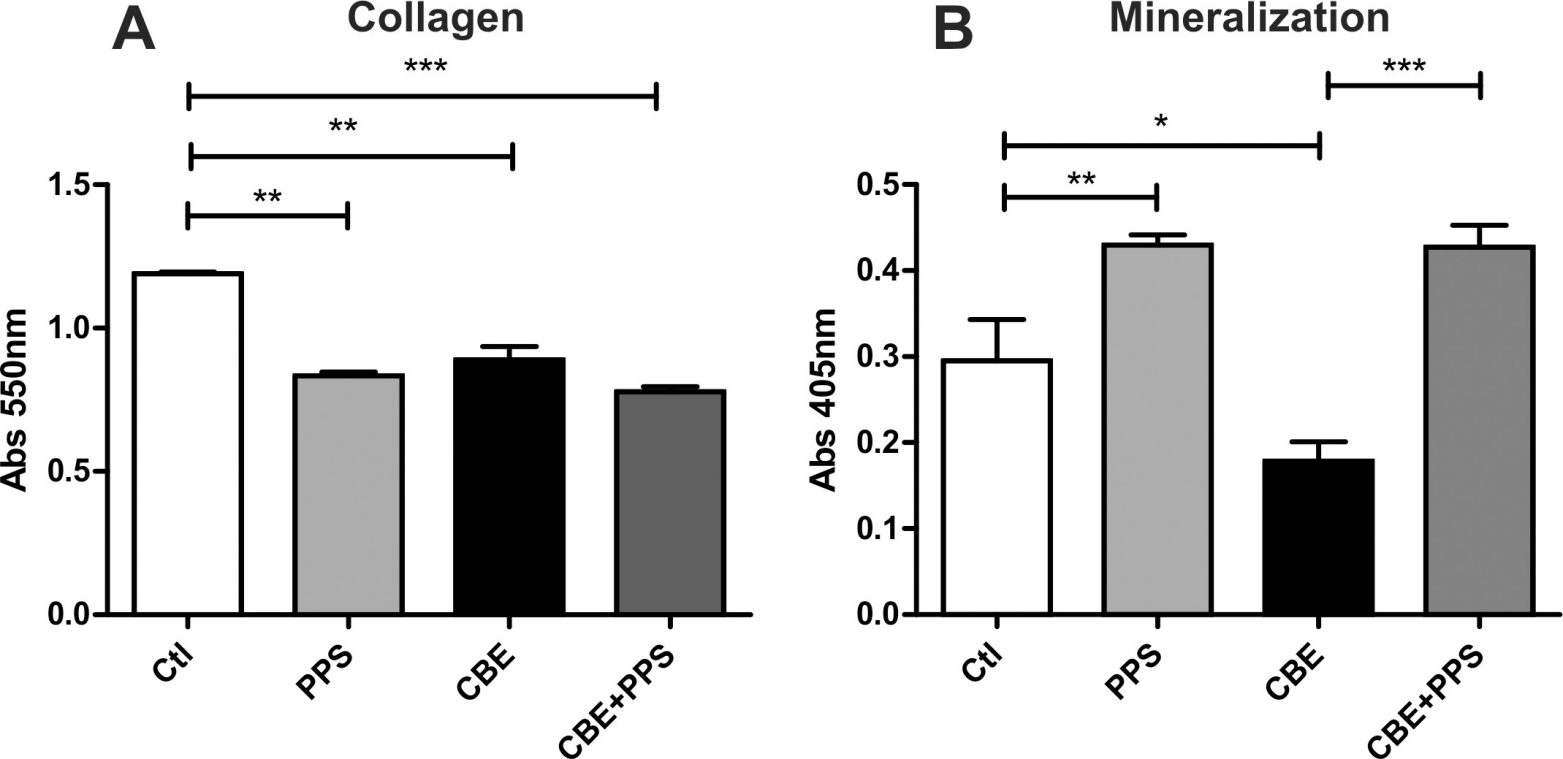
Bone formation activity as determined by collagen (A) and mineral deposition (B) by osteoblasts. Data is shown as the mean±SD with n = 5 one representative experiment of 3 performed. *p<0,05 **p<0,01 ***p<0,001 t-test.

The other side playing a role in bone homeostasis is bone formation mediated by osteoblasts. It has been proposed that osteoblast activity is reduced in GD [27]. To evaluate this effect, active osteoblasts were obtained from MC3T3 cells in the presence of CBE. CBE treatment reduced collagen deposition as well as mineralization (Fig 5A and 5B). Next we tested if PPS had any effect on these parameters. Osteoblasts were cultured in the presence of CBE and/or PPS. Mineral deposition was increased in PPS treated osteoblasts both in control and CBE cultures (Fig 5B), however surprisingly collagen deposition was reduced in the presence of PPS (Fig 5A).

## Discussion

Recent discoveries and developments in the field of lysosomal disorders have pointed our attention to physiopathological processes underlying these pathologies. Accumulation of deposits is simply and uniquely the first cell manifestation of damage, and is only one piece of the resultant pathogenesis in these diseases. This initial manifestation triggers the firing of multiple cascades of cellular mechanisms, being inflammation one of the most common and almost universal in lysosomal disorders. Chronic inflammation plays a critical role in tissue and organ malfunctioning [8].

Introduction of ERT was a revolution in the field and made a transformation for lives of patients afflicted with lysosomal disorders. However, existing ERT have proven incomplete correction of clinical affection [1]. Indeed, there is inconclusive evidence about the effect of ERT on inflammation. For this reason, adjuvant therapies directed to target inflammatory aspects appear as a rational option for treatment. In this regard, various studies have been carried out to prove that PPS is a useful treatment for different types of MPS, by its effect as an anti-inflammatory agent [15,17]).

Based on the knowledge that inflammation is a common pathological mechanism in lysosomal disorders together with the demonstrated anti-inflammatory effect of PPS in MPS we hypothesized PPS could be useful at reducing inflammation in other lysosomal disorders, such as Fabry and Gaucher.

There is no definitive conclusion as to whether ERT for Fabry disease modulates the immune system to reduce the level of inflammation. Some studies showed no statistical differences in the expression or the production of cytokines when comparing between naive patients or those undergoing ERT [4,28]. While other reports showed alterations of cytokine levels in patients on ERT. In this ambiguous context it may be valuable to find a compound able to reduce inflammatory components, as a possible way to improve/control the impact in target organs [29]. In this study, we were able to show that PPS reduces the secretion of TNFα and IL1β in a chemical *in vitro* model of Fabry disease to control levels. Moreover the same effect was seen in PBMC from Fabry patients. One of the main clinical symptoms in Fabry is the chronic and progressive nephropathy. This nephropathy has similarities to diabetic nephropathy (DN) [30]. In a recent model of DN TNFα was shown to contribute to the inflammatory lesions. PPS treatment in this model prevents the progression of nephropathy by decreasing albuminuria, renal macrophage infiltration and TNFα expression; along to improvement of histopathologic changes and renal function [31]. The heart is another main target organ in Fabry and PPS has been shown to improve cardiac function in different animal models [32,33]. One could speculate that PPS could have an effect in Fabry nephropathy and cardiomiopathy, and *in vivo* studies would be necessary to analyze this hypothesis.

Previous studies in Gaucher disease revealed Gaucher patients undergoing ERT display a proinflammatory profile, as demonstrated by increased production of proinflammatory cytokines by peripheral blood T cells and monocytes [12]. By the present work, we are able to affirm PPS, at least *in vitro*, has the capacity to reduce the secretion of those cytokines produced by PBMC.

In Gaucher disease skeletal problems are common and cause a high morbidity. As in other inflammatory syndromes, bone homeostasis is affected. The inflammatory condition induces higher bone resorption by osteoclasts and reduced bone formation by osteoblasts, resulting in low bone density [10]. It seems reasonable to think that a compound that reduces inflammation may have a positive impact in bone affection. For this reason we analyzed the use of PPS in *in vitro* bone models of Gaucher disease. In this study we detected that along with reduction of production of proinflammatory cytokines by PBMC, PPS was capable to reduce the tendency of osteoclast precursors to differentiate into active osteoclasts. In a recent article, it was demonstrated the molecular mechanism by which PPS inhibits osteoclastogenesis [34]. Moreover, when Gaucher models of PBMC, osteoblasts and osteocytes are exposed to PPS, their supernatant does not induce osteoclastogenesis implying that PPS downregulates the levels of pro-osteoclastogenic factors resulting in less of bone resorption. On the other hand, chitotriosidase has not changed with PPS treatment. It could be due to the reduced time of exposure of PPS of 3 days in the culture. Future experiments in animal models and/or human patients with longer exposure to PPS would elucidate if it has an effect.

Recently, PPS has been studied as a treatment for pain and lesions associated in osteoarthritis, a condition that also has an inflammatory profile [35]. The positive result in a bone inflammatory condition together with our results in the *in vitro* bone Gaucher disease model make PPS a potential drug to ameliorate skeletal problems in GD.

In order to discriminate if the effect of PPS was because of its well-known anti-inflammatory effect or because a yet unknown effect on glycolipid deposits we determined the levels of specific glycolipids for Fabry and Gaucher in cell homogenates and supernatants, with and without PPS. Incubation of cells with PPS for 3 days did not produce changes in glycolipids levels. By this work, we could rule out an effect of PPS on levels of deposits. It could also be possible that 3 days of incubation is a short time to view a change. Longer exposure to PPS on in vivo treatment could lead to reduction of glycolipids, like it was observed for GAGs reduction in MPS rat model [15]. Therefore the reduction of cytokine levels observed was not because reduction of deposits but because PPS acts directly as anti-inflammatory agent. In this context we could affirm PPS has a different mode of action independent of the one of specific therapies which its targets are glycolipids.

Importantly, our work confirms that PPS is effective not only in lysosomal disorders associated to GAGs accumulation but also for the ones with glycolipid deposits. It may possibly imply PPS could also be effective in lysosomal disorders associated to other accumulated substrates.

In conclusion, PPS is an effective molecule to reduce the production of proinflammatory cytokines in *in vitro* models of Fabry and Gaucher diseases. Moreover, it was effective at ameliorating bone alterations of *in vitro* models of Gaucher disease. This results serves as preclinical supportive data to start clinical trials in human patients to analyze the effect of PPS as a potential adjunctive therapy for Fabry and Gaucher diseases.
